# Role of abnormal glycosylated IgA1 and interstitial transformation of glomerular endothelial cells in the development and progression of IgA nephropathy

**DOI:** 10.1186/s13052-023-01468-x

**Published:** 2023-05-11

**Authors:** Wanyu Jia, Wenjie Dou, Qin Wang, Huiqin Zeng, Peipei Shi, Jing Liu, Zhen Liu, Jin Zhang, Jian-Jiang Zhang

**Affiliations:** 1 Department of Pediatrics, Clinical Center of Pediatric Nephrology of Henan Province, the First Affiliated Hospital of Zhengzhou University, No. 1 Jianshe East Road, Erqi District, Zhengzhou, 450052 Henan Province China; 2 Department of Nephrology, Shanghai Tongji Hospital, Tongji University School of Medicine, Shanghai, China

**Keywords:** IgA nephropathy, Abnormally glycosylated IgA1, Endothelial cell interstitial transformation, Renal fibrosis, Pathogenesis

## Abstract

**Background:**

IgA nephropathy (IgAN) is a common primary renal disease in childhood.

**Methods:**

Twenty blood samples and renal tissue from patients with IgAN, 20 blood samples from healthy children and 10 normal renal tissue were collected. Serum Gd-IgA1 and renal Gd-IgA1, CD31, α-SMA and vimentin were measured.

**Results:**

The serum Gd-IgA1 concentration in the IgAN group was significantly higher. Gd-IgA1 was not expressed in normal kidneys, which was positive in the IgAN group. Gd-IgA1 levels in serum and renal tissue were not related. The expression of CD31 decreased significantly in IgAN group, while the expression of α-SMA and vimentin increased significantly. There was no significant correlation between the renal concentration of Gd-IgA1 and CD31, α-SMA and vimentin.

**Conclusion:**

The increased Gd-IgA1 in the serum and kidney may promote the pathogenesis of IgAN. The serum Gd-IgA1 cannot predict the extent of its deposition in the kidney. Endothelial mesenchymal transition (EndMT) may be involved in the pathogenesis of renal fibrosis in IgAN.

## Background

IgA nephropathy is one of the most common primary glomerular diseases in childhood. The main immunopathological feature of IgA nephropathy (IgAN) is the deposition of IgA in the mesangial region. IgA nephropathy is a chronic progressive disease and is one of the main glomerular diseases clinically leading to end-stage kidney disease and chronic renal failure [1]. Approximately 25–30% of patients with IgA nephropathy develop varying degrees of chronic kidney disease (CKD) within 20 to 25 years after the disease, requiring dialysis or kidney transplantation. The epidemiology, clinical manifestations, disease progression, and prognosis of IgA nephropathy vary among ethnic populations around the world. IgAN is most prevalent in Asian populations, more likely to cause kidney failure, followed by Caucasians and relatively rare in populations of African ancestry [1]. In China, the incidence among the IgA nephropathy ranks first in the incidence of primary glomerulonephritis for many years [2]. At present, the etiology and pathogenesis of IgA nephropathy have not been fully elucidated.

IgA deposition in the kidney is not only an immunopathological feature of IgAN but also an important factor involved in renal injury. IgA molecules in the human body are highly glycosylated immunoglobulins, including IgA1 and IgA2. IgA molecules exist in the human body in the form of monomers (90%) and multimers, and the main molecule deposited in the kidney of IgAN is multimer IgA1 [3]. A large number of studies have confirmed that there is abnormally glycosylated IgA1 (galactose deficient IgA1, Gd-IgA1) in the serum and urine of IgAN patients, which is significantly higher than that of other glomerular diseases, such as nephrotic syndrome, lupus nephritis and other patients and healthy controls crowds [4, 5]. Most scholars believe that Gd-IgA1 plays an important role in the pathogenesis and disease progression of IgAN [6, 7].

Renal fibrosis is a common pathway for a variety of renal diseases to gradually aggravate and develop to different degrees of kidney failure. Excessive proliferation of myofibroblasts in the process of renal fibrosis has a wide range of sources, and a large number of studies have confirmed that endothelial to mesenchymal transition (EMT) is involved in the occurrence and development of renal fibrosis like in other organs [8]. Recent studies have shown that endothelial cells can also transform to mesenchymal cells (endothelial-to-mesenchymal transition, EndMT) and play an important role in the process of fibrosis [9–11]. Endothelial cells that develop EndMT lose the expression of endothelial cell-specific proteins, such as vascular endothelial cadherin (VE-cadherin) and CD31/platelet endothelial cell adhesion molecule (PECAM-1), and initiate the expression of mesenchymal cell-specific genes. Expression and synthesis of encoded proteins, such as α-smooth muscle actin (α-SMA), fibroblast specific protein-1(FSP-1), extra domain A (EDA) fibronectin and vimentin [12]. Studies have demonstrated that EndMT plays a role in renal fibrosis in some renal diseases. Zeisberg et al. had confirmed the existence of EndMT in mouse models of obstructive nephropathy, diabetic nephropathy, and Alport syndrome [13]. Later, Li et al. confirmed the existence of endothelial cell-derived α-SMA + fibroblasts in diabetic nephropathy model [14]. EndMT was subsequently shown to promote peripheral vascular fibrosis in patients with type 2 diabetes and in a mouse model of diabetes (induced by streptozotocin) [15]. Patients with IgA nephropathy will have different degrees of renal failure within a few years after the onset, and the development of fibrosis has always been the focus of their research. To date, there are no related studies of EndMT in IgAN. Therefore, our objective was to speculate whether EndMT occurs in the kidneys of patients with IgA nephropathy.

In this study, we detected the levels of Gd-IgA1 in the blood of children with IgAN and the expression of Gd-IgA1, CD31, α-SMA, and vimentin in the kidneys to explore the level of Gd-IgA1 in the blood and kidneys of children with IgAN. The occurrence of endothelial cell-mesenchymal transition in renal glomerular cells, and their roles in IgAN, may provide new directions and targets for the clinical treatment of IgAN.

## Methods

### Patients

This retrospective study, consisted of children with IgA nephropathy, admitted to the Pediatrics Department of the First Affiliated Hospital of Zhengzhou University between October 2019 and September 2021 with complete clinical data were included as the IgA nephropathy group. The inclusion and exclusion criteria were as follows: (1) Age group form1 -18 years; (2) Histopathologic features consistent with the diagnostic criteria of pediatric primary IgA nephropathy [16], and the diagnosis was confirmed as primary IgA nephropathy; (3) Children with secondary IgA nephropathy, such as IgA vasculitis, systemic lupus erythematosus (SLE) and other vasculitic rheumatic diseases, were excluded; (4) Patients with liver and kidney function damage secondary to systemic diseases, such as diabetes were excluded. (5) Children who were treated with glucocorticoids and other immunosuppressants, such as tacrolimus and cyclosporine were excluded.

So, we included 20 patients in IgA nephropathy group, 20 healthy children who underwent physical examination during the same period as the serum control group. Ten cases of normal unaffected renal tissue adjacent to the kidney tumor of the children who underwent radical nephrectomy in the Pediatric Urology Department of our hospital were used as the kidney control group.

### Sample collection

Demographic data and baseline characteristics of patients were collected at the time of admission. Two milliliters of venous blood were taken from all subjects in the morning, and the supernatant was taken and stored in a -80 refrigerator for later use. Renal tissue of children with IgA nephropathy was collected by puncture and embedded in paraffin. The normal renal tissue was derived from the uninvolved adjacent tissue of children with renal tumors, and the tissue was frozen in liquid nitrogen after surgery.

### Measurement of serum Gd-IgA1 by ELISA

The Gd-IgA1 level in serum was measured using a solid phase sandwich ELISA test (galactose-deficient IgA1 assay kit, Immuno-Biological Laboratories Co., Ltd, Japan).

Wells were used to determine the test sample blank. A total of 50 µl of each EIA buffer was placed into the wells. We placed 50 µl of the prepared test samples and prepared standards into appropriate wells. Incubation with plate lid. Wash the plate with the prepared wash buffer and remove all liquid. Then, 50 µl of prepared labeled antibody was placed into the wells. Incubation with plate lid. The plate was washed with the prepared wash buffer, and all liquid was completely removed. Add 50 µl Chromogen - tetramethyl benzidine (TMB) solution. Incubation in the dark. Add 50 µl stop solution. Remove any dirt or drop of water on the bottom of the plate. Then, both the optical density of the standard and the test samples were measured against a test sample blank. Measurement wavelength: 450 nm. All the samples were tested in duplicate, and the mean values were used for analysis.

### Measurement of Gd-IgA1, CD31, α-SMA, and vimentin by immunohistochemistry

Paraffin-embedded sections of 4-µm thickness were prepared for staining. After deparaffinization by a series of xylene/ethanol and rehydration, the sections for Gd-IgA1 staining were treated with 0.4% pepsin (Servicebio, Wuhan, China) for 25 min for antigen retrieval. The sections for CD31, α-SMA, and vimentin staining were heated with Citrate Antigen Retrieval Solution (Servicebio, Wuhan, China) in a microwave oven for antigen retrieval. Endogenous peroxidase was blocked by reacting with peroxide solution for 25 min at room temperature in the dark. Blocking with bovine albumin was performed at room temperature for 30 min. The cells were incubated with KM55 [17] (Immuno-Biological Laboratories Co., Ltd, Japan) (1:20), polyclonal rabbit anti-human α-SMA (Servicebio, Wuhan, China) (1:500), polyclonal rabbit anti-human CD31 (Servicebio, Wuhan, China) (1:300) and polyclonal rabbit anti-human vimentin (Bioss, Beijing, China) (1:300) overnight at 4 °C. After washing, the secondary antibody of the corresponding species was added and incubated at room temperature for 50 min. DAB staining solution was used for staining, and the color was brown to indicate positive staining. After washing with PBS, slides were sealed in neutral balsam mounting medium. The sections were examined by light microscopy (Nikon, Japan). Five fields were randomly selected from each section for imaging. Image-Pro Plus 6.0 analysis software was used to analyze the imaging. Unit pixels were taken as the standard unit, and the same brown‒yellow color was selected as the unified standard to judge the positive part of all photos. Each image was analyzed to obtain the cumulative optical density (IOD) of positive expression in the glomerular and tubulointerstitium region and the selected glomerular and tubulointerstitium pixel area, respectively. Finally, Areal density = IOD/glomerular area was calculated, and the average value of the five fields was taken.

### Statistical analysis

SPSS 26.0 statistical software was used for statistical analysis of the experimental data. The Kolmogorov‒Smirnov test was used to test the normality of the experimental data. Normally distributed data are expressed as the mean and standard deviation, and nonnormally distributed data are expressed as the median and interquad M (P25, P75). T test was used for comparison when normal distribution data met homogeneity of variance; Kruskal‒Wallis nonparametric test was used for correlation analysis when data did not meet the above conditions; Pearson correlation analysis was used when data met normal distribution; Spearman correlation analysis was used for data that did not meet normal distribution. P<0.05 means the difference is statistically significant.

This study was approved by the Medical Ethics Committee of the First Affiliated Hospital of Zhengzhou University (2021-KY-1221-002). All subjects signed the consent form before participation in the study. The study was conducted in accordance with the Declaration of Helsinki (as revised in 2013).

## Result

### Baseline characteristics

There were 20 cases in the IgA nephropathy group, including 14 males and 6 females, and the age at the time of renal biopsy was 8.5 (7.0, 11.0) years. There was no significant difference in age or sex between the IgA nephropathy group and the serum control group. There was no significant sex difference between the IgA nephropathy group and the kidney control group. Routine urine test results were normal for both serum control group and renal control group, with no proteinuria or urinary red blood cells. Serum creatinine (SCr) and estimated glomerular filtration rate (eGFR) were slightly higher in the IgA nephrotic group than in the control groups(P<0.05), but both were within the normal range, and this difference may be related to the age of the children. And the levels of serum uric acid (SUA), blood urea nitrogen (BUN), total cholesterol (TC), triacylglycerol (TG), high-density lipoprotein-cholesterol (HDL-C), and high-density lipoprotein-cholesterol (LDL-C) were not different between the IgA nephropathy and control groups and were all within the normal range(P>0.05). The MEST scores of the IgA nephropathy group were shown in Table 1.

**Table 1:** The clinical baseline information of the IgA nephropathy group, serum control group and kidney control group

		IgA nephropathy group	serum control group	kidney control group
Sex (Male/Female)		14/6	11/9	4/6
Age (year)		8.5(7.0,11.0)	9.5(9.0,13.0)	2.5(2.0,5.3)
urine protein (g/24 h)		1.2 ± 0.25	0	0
urine erythrocyte(/µL)		382.5(75.8,996.3)	0	0
SCr (µmol/L)		51.9 ± 18.4	43.9 ± 10.2	32.7 ± 13.8
eGFR (ml/min)		229.9 ± 81.3	132.8 ± 28.0	78.8 ± 19.8
SUA (µmol/L)		272.5 ± 73.4	262.6 ± 81.2	335.1 ± 147.2
BUN (mmol/L)		4.3 ± 1.4	4.6 ± 1.2	4.7 ± 1.3
TC (mmol/L)		4.6 ± 1.6	3.8 ± 0.8	/
TG (mmol/L)		1.2 ± 0.6	0.9 ± 0.4	/
HDL-C(mmol/L)		1.4 ± 0.2	1.4 ± 0.3	/
LDL-C(mmol/L)		2.8 ± 1.4	2.1 ± 0.7	/
Serum IgA (g/L)		2.5 ± 0.063	/	/
C3(g/L)		1.2 ± 0.2	/	/
systolic pressure(mmHg)		103(98.5,107.5)	/	/
diastolic pressure(mmHg)		65(60,65)	/	/
MEST score	M0/M1	8/12	/	/
	S0/S1	7/13	/	/
	T0/T1	13/7	/	/
	E0/E1	20/0	/	/

### Serum levels of Gd-IgA1

**Fig. 1:**
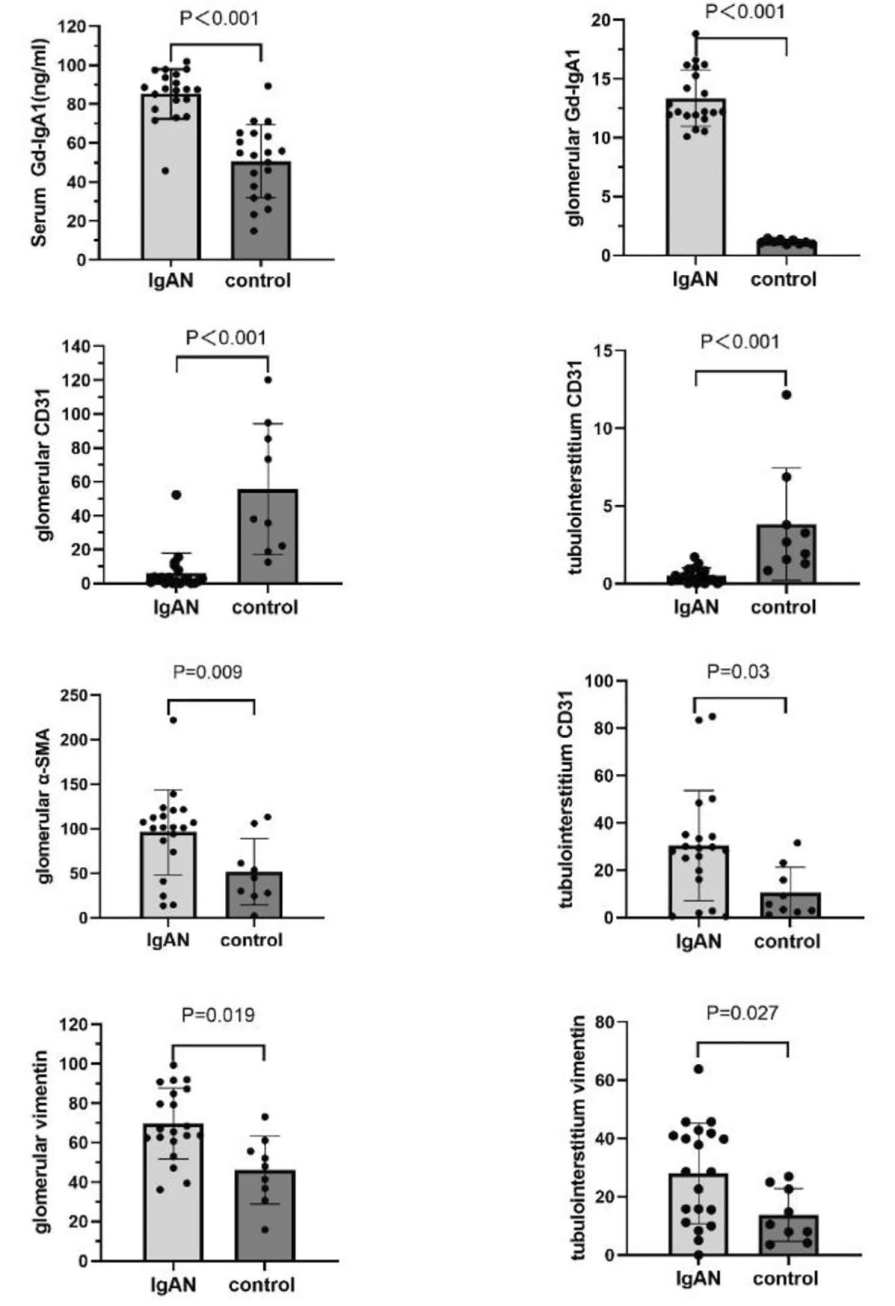
Serum levels of Gd-IgA1 and areal density of Gd-IgA1, CD31, α-SMA and vimentin in the kidney

The concentration of abnormal glycosylated IgA1 in the serum of the IgA nephropathy group was 85.2 ± 12.8 ng/ml, which was 50.6 ± 18.7 ng/ml in the serum control group. The concentration of abnormal glycosylated IgA1 in the serum of the IgA nephropathy group was significantly higher than that of the serum control group, and the difference was statistically significant (P < 0.05). (Fig. 1)

### The deposition Gd-IgA1 and expression of CD31, α-SMA and vimentin in the kidney

**Fig. 2:**
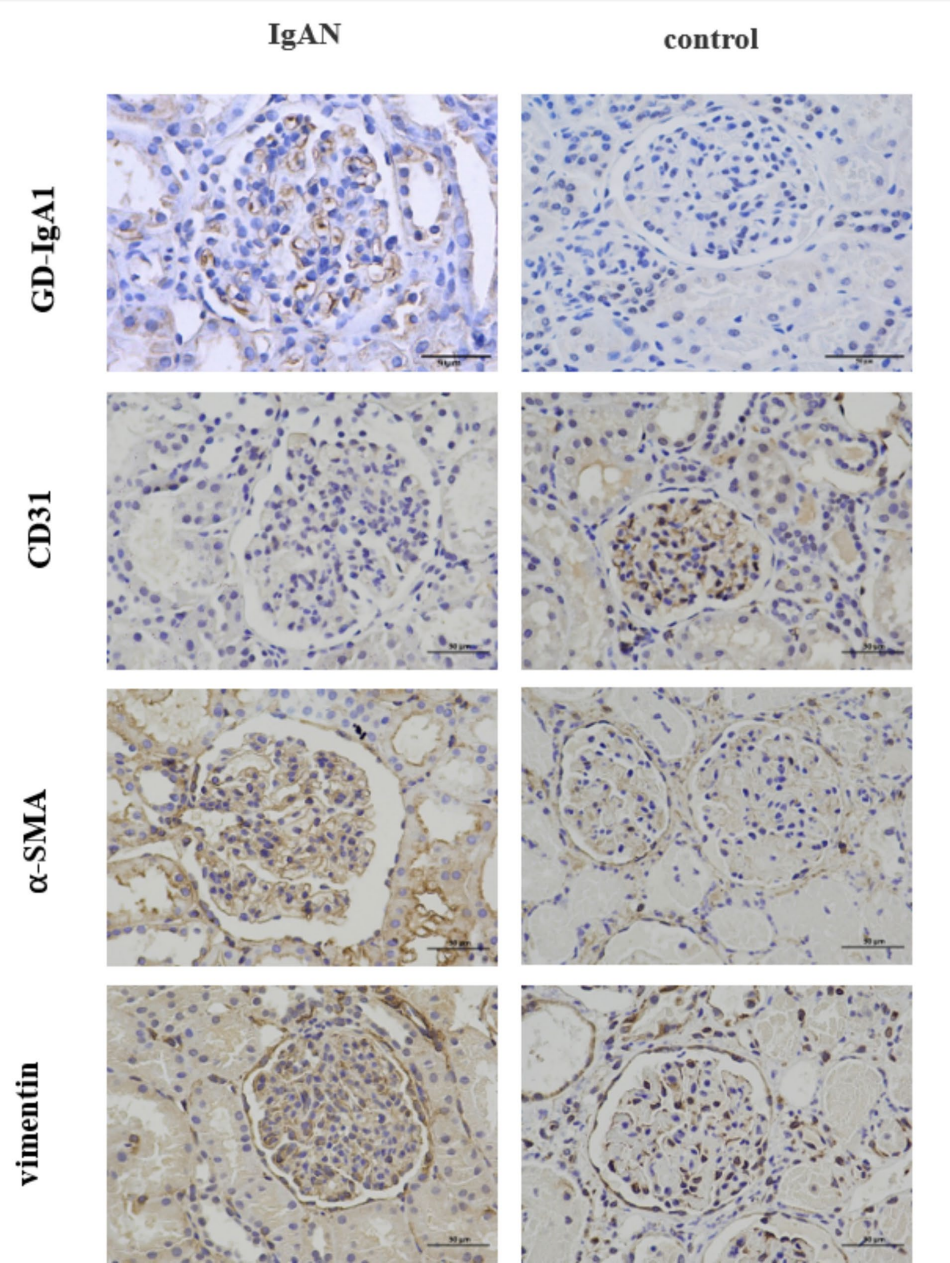
Expression of Gd-IgA1, CD31, α-SMA and vimentin in the kidney (×400)

The abnormal glycosylation IgA1 staining in the kidneys of the children in the kidney control group was negative, while the IgA nephropathy group showed positive expression of abnormal glycosylation IgA1 in the glomerulus, which was mainly found in the mesangial cells. The expression of CD31 in the IgA nephropathy group was significantly lower than that in the control group, which was mainly expressed in the glomerulus and rare in the renal tubules. α-SMA was expressed at lower levels in the kidney control group, mainly in the glomerulus. There was a large amount of α-SMA expression on the glomerulus and renal tubule in the IgA nephropathy group. Compared with the kidney control group, the expression of vimentin on the glomerulus and renal tubule of the IgA nephropathy group were both significantly increased. (Fig. 2)

The areal density of CD31 in the glomerulus of the IgA nephropathy group was significantly decreased both in glomerulus and tubulointerstitium regions (P < 0.05). The areal density of α-SMA and vimentin was significantly increased (P < 0.05).

### Correlation analysis

The correlation analysis of serum abnormal glycosylated IgA1 concentration and renal abnormal glycosylated IgA1 expression (surface density) showed that there was no significant correlation between the two (P > 0.05). The Serum ratio of Gd-IgA1/C3 in the IgA nephropathy group did not correlate significantly with abnormal glycosylated IgA1 expression (P > 0.05).

**Fig. 3:**
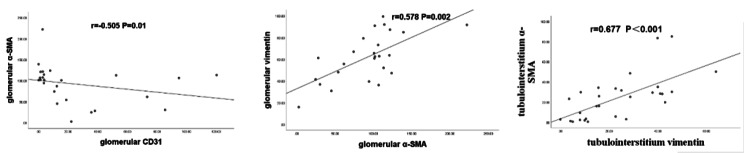
Correlation of expression of CD31, α-SMA and vimentin

In the IgA nephropathy group, there was no significant correlation between the expression of Gd-IgA1 in the kidney and the expression of CD31, α-SMA and vimentin both in glomerulus and tubulointerstitium regions (P > 0.05) (Table 2).Meanwhile, there was a significant negative correlation between glomerular CD31 surface density and glomerular α-SMA surface density (r=-0.505 P = 0.01) and a significant positive correlation between glomerular α-SMA surface density and glomerular vimentin surface density (r = 0.578 P = 0.002). And there was a significant positive correlation between the expression of tubulointerstitium α-SMA and tubulointerstitium vimentin in the glomerular region (r = 0.677 P<0.001). (Fig. 3).

**Table 2:** Correlation of expression of Gd-IgA1 and the expression of CD31, α-SMA and vimentin in the kidney

Gd-IgA1	r	P
glomerular CD31	0.133	0.681
Glomerular α-SMA	-0.427	0.167
glomerular vimentin	0.182	0.572
tubulointerstitium CD31	-0.258	0.394
tubulointerstitium α-SMA	-0.33	0.271
tubulointerstitium vimentin	-0.17	0.578

## Discussion

IgA nephropathy is one of the most common primary glomerular diseases in childhood in China [1]. Studies have confirmed that Gd-IgA1 molecules in patients with IgA nephropathy are significantly higher than those in healthy people and patients with other glomerular diseases, such as nephrotic syndrome and lupus nephritis, and are deposited in the glomerular region of patients with IgA nephropathy [18, 19]. This study confirmed that the serum Gd-IgA1 concentration in children with IgA nephropathy was significantly higher than that in healthy children. At the same time, the kidneys of children with IgA nephropathy have obvious Gd-IgA1 deposition, which is mainly deposited in mesangial cells. This is consistent with previous research results, suggesting that Gd-IgA1 may promote the occurrence and development of IgA nephropathy. Current research suggests that Gd-IgA1 is involved in the occurrence and development of IgAN in various ways. In this study, there was no significant correlation between the abnormal glycosylation of IgA1 in the serum and kidney of children with IgA nephropathy. And although Chen P et al. [20] had confirmed a linear correlation between serum Gd-IgA1/C3 levels and the CKD progression of IgAN, our study found no direct correlation between serum Gd-IgA1/C3 levels and Gd-IgA1 deposition. These suggested that the deposition of Gd-IgA1 molecules in the kidney in IgA nephropathy may not only be affected by its circulating concentration and it is possible that Gd-IgA1 are not only deposited directly onto the glomerulus, but that more complex mechanisms are involved that need to be investigated. The serum concentration of abnormal glycosylated IgA1 and Gd-IgA1/C3 cannot predict the extent of its deposition in the kidney.

EndMT has been confirmed to be involved in the occurrence and development of various kidney diseases. In recent years, studies have confirmed that EndMT exists in diabetic nephropathy from different aspects, contributes to the formation of fibroblasts and participates in the occurrence and development of early renal fibrosis and glomerulosclerosis [21–23]. In this study, CD31, α-SMA, and vimentin were selected as markers of EndMT, and the expression of CD31 in the glomerulus of children with IgA nephropathy was significantly decreased, while the expression of α-SMA and vimentin was significantly increased. And we found that the expression of α-SMA and vimentin there was a significant positive correlation between α-SMA and vimentin expression in kidney, and a significant negative correlation between SMA and CD31 expression in glomerulus. The results of this study confirm that EndMT occurs in both glomerular and tubular epithelium in children with IgA nephropathy. the occurrence of EndMT may be one of the steps in the fibrotic process of IGA nephropathy. It can be speculated that the process of endothelial cell to mesenchymal cell transition is involved in IgA in the pathogenesis of kidney disease.

In this study, we found no correlation between the expression of Gd-IgA1 and EndMT related proteins (CD31, -SMA, and vimentin). We speculated that this result might be connected to the children we included having milder clinical and pathological presentations. Children with IgA nephropathy in our included group were diagnosed early and in the early stages of disease progression, exhibited minimal renal fibrosis. Long-term follow-up of children in this group may lead to different outcomes. Future research may be required to confirm the relationship between Gd-IgA1 and EndMT in patients with IgA nephropathy who are suffering from the disease’s latter stages. Studies have confirmed that Gd-IgA1 can promote the synthesis and secretion of TGF-β1 in renal mesangial cells and podocytes in patients with IgA nephropathy [24, 25]. At present, most scholars believe that TGF-β plays an important role in stimulating the progression of EndMT and is one of the initiating factors of EndMT [12, 26]. Therefore, we can speculate that Gd-IgA1 molecules deposited in renal mesangial cells may induce the occur of EndMT in glomerular and tubular endothelial cells by stimulating increased secretion of TGF-β from renal mesangial cells and podocytes. The deposition of Gd-IgA1 and occur of EndMT in kidney may be involved in the development and progression of IgA nephropathy in a synergistic manner.

There are also some shortcomings in this study: (1) This experiment is a single-center retrospective study with a small sample size, and the experimental results still need to be further confirmed by a larger sample size study. (2) There is no further study on the relationship between TGF-β and abnormal glycosylation. The relationship between IgA1 renal deposition and EndMT in glomerular epithelial cells. (3) The relationship between the occurrence of EndMT and the severity and prognosis of IgA nephropathy was not further explored. More in-depth studies are needed to explore the mechanism of aberrantly glycosylated IgA1 and glomerular endothelial cell-mesenchymal transition in the occurrence and development of IgA nephropathy. This study is the first to confirm the occurrence of interstitial transition of glomerular endothelial cells in IgA nephropathy, which provides a new idea for further research on the pathogenesis of IgA nephropathy.

## Conclusion

The levels of Gd-IgA1 in the serum and kidney of children with IgA nephropathy are increased, and the increased level of abnormal glycosylated IgA1 in the serum and kidney in IgA nephropathy may promote the occurrence and development of IgA nephropathy. The serum concentration of abnormal glycosylated IgA1 cannot predict the extent of its deposition in the kidney. Endothelial cell interstitial transformation exists on the glomerulus of children with IgA nephropathy. Further research on EMT, the mechanism of sclerosis (kidney fibrosis) still needed.

## Data Availability

The datasets used and/or analysed during the current study are available from the corresponding author on reasonable request.

## List of Abbreviations

α-SMA: α-smooth muscle actin
BUN: Blood urea nitrogen
CKD: Acute renal failure
EDA: Extra domain A
eGFR: Estimated glomerular filtration rate
EMT: Endothelial to mesenchymal Transition
EndMT: Endothelial-to-mesenchymal transition
FSP-1: Fibroblast specific protein-1
Gd-IgA1: Abnormally glycosylated IgA1
HDL-C: High-density lipoprotein-cholesterol
HSP: Allergic purpura
IgAN: IgA nephropathy
IL-6: Interleukin-6
IOD: Obtain the cumulative optical density
LDL-C: High-density lipoprotein-cholesterol
PECAM-1: Platelet endothelial cell adhesion molecule
SCr: Serum creatinine
SLE: Systemic lupus erythematosus
SUA: Serum uric acid
TC: Total cholesterol
TG: Triacylglycerol
TGF-β: Transforming growth factor-β
TMB: Tetramethyl benzidine
TNF-α: Tumor necrosis factor-α
VE-cadherin: Vascular endothelial cadherin
